# Modelling the probability of erroneous negative lymph node staging in patients with colon cancer

**DOI:** 10.1186/s40880-019-0377-5

**Published:** 2019-06-06

**Authors:** Carlos Fortea-Sanchis, Erica Forcadell-Comes, David Martínez-Ramos, Javier Escrig-Sos

**Affiliations:** 1 Division of Colorectal Surgery, Department of Surgery, Consorci Hospitalari Provincial de Castelló, Castelló, Spain; 2 Oropesa Health Center, Carrer de Torreblanca, 14, 12594 Oropesa, Castelló Spain; 3 Department of Surgery, Hospital General de Castelló, Castelló, Spain

**Keywords:** Colon cancer, Lymph node staging, Bayes’ theorem, Final error probability, Prognosis

## Abstract

**Background:**

Patients in who with insufficient number of analysed lymph nodes (LNs) are more likely to receive an incorrect LN staging. The ability to calculate the overall probability of undiagnosed LN involvement errors in these patients could be very useful for approximating the real patient prognosis and for giving possible indications for adjuvant treatments. The objective of this work was to establish the predictive capacity and prognostic discriminative ability of the final error probability (FEP) among patients with colon cancer and with a potentially incorrectly-staged LN-negative disease.

**Methods:**

This was a retrospective multicentric population study carried out between January 2004 and December 2007. We used a mathematical model based on Bayes’ theorem to calculate the probability of LN involvement given a FEP test result. Cumulative sum graphs were used to calculate risk groups and the survival rates were calculated, by month, using the Kaplan–Meier method.

**Results:**

A total of 548 patients were analysed and classified into three risk groups according to their FEP score: low-risk (FEP < 2%), intermediate-risk (FEP 2%–15%), and high-risk (FEP > 15%). Patients with LN involvement had the lowest overall survival rate when compared to the three risk groups. This difference was statistically significant for the low- and intermediate-risk groups (*P* = 0.002 and *P* = 0.004, respectively), but high-risk group presented similar survival curves to pN+ group (*P* = 0.505). In terms of disease-free survival, the high-risk group presented similar curves to the intermediate-risk group until approximately 60 months’ follow-up (*P* = 0.906). After 80 months’ follow-up, the curve of high-risk group coincided with that of the pN+ group (P = 0.172). Finally, we summarized the FEP according to the number of analysed LNs and accompanied by a contour plot which represents its calculation graphically.

**Conclusions:**

The application of Bayes’ theorem in the calculation of FEP is useful to delimit risk subgroups from among patients without LN involvement.

## Introduction

Colon cancer is the most frequent malignancy in both sexes in Western countries, with an incidence of approximately 471,000 cases per year and a mortality of 228,000 cases per year in Europe [1]. Lymph node (LN) involvement is the prognostic factor most directly related to the survival and disease-free interval of cancer patients. Thus, patients with stages I and II cancers have a 5-year overall survival (OS) rate higher than 75% compared to 30%–60% in patients with stages III and IV cancers [2–4]. Tumor-node-metastasis (TNM) classification is the gold standard method for staging colon cancer, however, this system recommends collecting at least 12 LNs for correct staging. Despite the multidisciplinary approach to LN analysis in colon cancer, for various reasons related to patients, surgeons, and pathologists, the number of LNs analysed is very variable between patients [5], and significantly fewer than 12 are usually analysed [6, 7]. Without a doubt, patients with a pN0 LN staging have the highest risk (in terms of their therapeutic management) of suffering the most harmful consequences of being given an incorrect classification and prognosis. Calculation of the final error probability (FEP), i.e. the probability that the patients will present undiagnosed LN involvement, would be very useful for predicting the real prognosis and possible indications for adjuvant treatment among these patients.

The Bayes’ theorem statistical method can be used to calculate the probability of presenting affected LNs even when a patient presents negative anatomopathological study results. It considers the anatomopathological study of the surgical specimen as a diagnostic test with a binary result in this case: positive LNs (presence of disease) or negative LNs (absence of disease) [8]. The probability of a patient having the disease, even when given a negative test result (i.e., the probability that a patient with a negative histological study result has an unidentified lymph-node metastasis) is represented by the complementary value of the final negative predictive value. The objective of this work was to establish the predictive capacity and prognostic discriminative ability of the FEP among patients with colon cancer and with a potentially incorrectly-staged LN-negative disease.

## Materials and methods

### Patients

This is a multicentric population study using data from a high-quality tumour sample registry included in the European cancer registry-based study on survival and care of cancer patients (EUROCARE study) [1]. The data used from this registry corresponded to the period between January 2004 and December 2007. Patients with colon cancer treated with surgery with curative intent and lymphadenectomy, a complete anatomopathological report, and a clear clinical status at their last follow up were included. Patients with cancer of the rectum or caecal appendix, with metastases at diagnosis, scheduled surgery with palliative intention without lymphadenectomy, scheduled surgery without resection, incomplete anatomopathological reports, a dubious vital status at the last follow-up control, and those with insufficient or no monitoring were excluded. The study was approved by the institutional review board of the Hospital General de Castellon (PIC: 2013/2/CIR). All participating patients provided their written informed consent.

### Variables

The study variables were age, sex, tumour location, histology, differentiation grade, and the size, number of analysed LNs, number of positive LNs, TNM classification, condensed T and N stages, chemotherapy, FEP, OS, disease-free survival (DFS), overall recurrence, locoregional recurrence, metastasis, and follow-up time.

Because all the data in the tumour registry is coded according to the sixth edition of the Union for International Cancer Control (UICC) TNM classification, we had to adapt them to the new guidelines for the seventh edition. Thus, although the N category was easily adapted, the T category could not be adapted to the new classification because the tumour registry contained insufficient data. As in other population studies, to minimise the effects of possible misclassifications, we used condensed TNM stages. The recurrence variable included patients who presented locoregional recurrence and those who presented distant metastases.

### FEP

We used a well-known mathematical model based on Bayes’ theorem to calculate the various diagnostic test parameters (sensitivity, specificity, and predictive values). According to Bayes’ theorem, the FEP is the probability of LN involvement (N+) given a negative test result (n−), in other words, p(N+/n−), can be deduced from the following mathematical formula [8]:

$$p(N + \left| {n- } \right.) = \frac{{p(N+)*p(n - \left| {N+} \right.)}}{{\left[ {p(N+)*p(n - \left| {N+} \right.)} \right] + \left[ {p(N- )*p(n - \left| {N- } \right.)} \right]}}$$

In the Bayes’ theorem formula: p(N+) is the prevalence of pN1 cases in the series; p(N−) is the complement to p(N+); p(n−/N+) is probability of a false negative (1 − Sensitivity) and is calculated by obtaining the hypergeometric probability resulting from the consideration of (1) the total LNs analysed from all the patients in the series; (2) the total number of positive LNs obtained in the series; (3) the number of positive LNs in a specific patient (equal to 0 for pN0 cases); and (4) the number of LNs analysed in a specific patient; the Specificity is p(n−/N+) and equals 1 because the presence of false ganglionic positives is considered impossible.

Given that there is a substantially greater probability of patients misclassified as pN0 (because they had an insufficient number of LNs analysed) having pN1 rather than pN2 or pN3 tumours, we decided to calculate the FEP of pN1 incorrectly being classified as pN0. Thus, all the FEP calculations refer to this adjusted FEP, set to pN1. Once the FEP was obtained, Cumulative Sum (CUSUM) [9] curves were used to calculate the optimal cut-off points following the method described by Barrio et al. [10], to obtain three incorrect pN0 classification risk groups.

### Statistical analysis

Quantitative variables are expressed as the mean ± standard deviation (SD). Categorical variables are reported as frequencies and percentages. For the univariate analysis, the Chi square test (or exact Fisher test in small samples) was used to compare two qualitative samples; the Student t-test was used to compare two quantitative samples; and the ANOVA test was used to compare more than two quantitative samples.

The follow-up time we considered was from the date of surgery until the day of death, or the last day of follow-up in patients who did not die. This was because the tumour registry did not contain any clear definition of the date of diagnosis. Survival analysis was performed using the Kaplan–Meier method and the log-rank test was implemented to estimate the differences between groups in terms of OS and DFS. Probability values of *P *< 0.05 were accepted as the statistical significance cut-off level. Statistical analysis was carried out with the IBM SPSS Statistics^®^ program version 22 (IBM^®^, Armonk, New York, USA). The CUSUM curves were calculated using the STATA^®^ program version 14 (StataCorp LP^®^, College Station, Texas, USA).

## Results

**Fig. 1:**
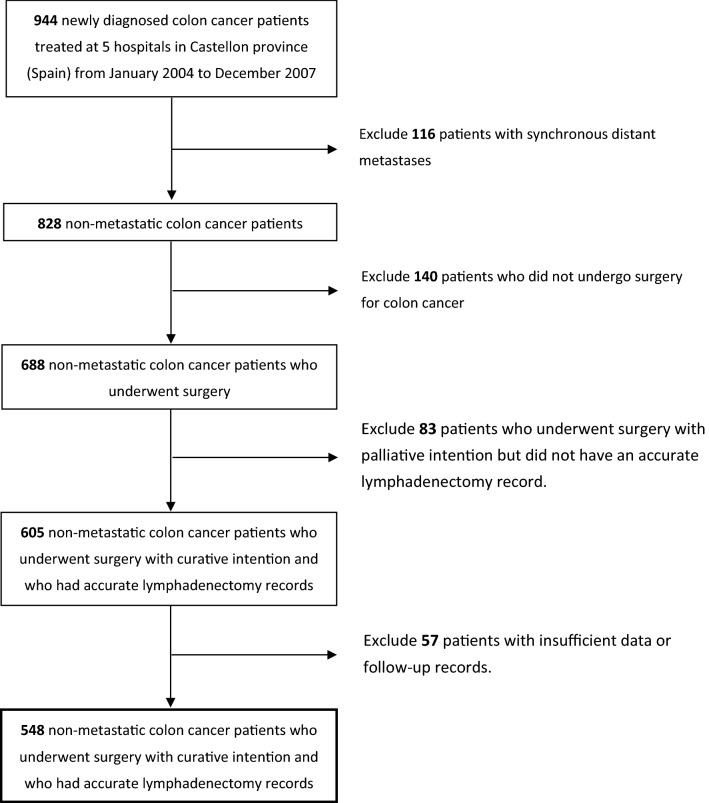
Enrolment details of the present study participants

During the period from January 2004 to December 2007, 944 patients were diagnosed with colon cancer in Castellon province (Spain), 140 of which were not operated on because they had contraindications for anaesthesia or because they had unresectable neoplasms. Eighty-three patients were operated on with palliative intention and did not have an accurate lymphadenectomy record and so were not included in this work. We also excluded 116 cases with colon neoplasms that did receive an intervention but who also presented synchronous distant metastases. Insufficient data were obtained regarding the number of LNs analysed and affected in 49 cases and for 8 of the patients, there were no follow-up data recorded. Thus, here we eventually analysed 548 patients (Fig. 1).

According to the adjusted FEP and the optimal cut-off points, the low-risk (FEP < 2%) category corresponded to patients who had > 20 analysed LNs; the intermediate-risk (FEP 2%–15%) corresponded to cases with 6–20 analysed LNs; and high-risk (FEP > 15%) corresponded to patients with ≤ 5 analysed LNs.

The clinical and histopathological characteristics of the 548 patients, and of the three risk-groups, are shown in Table 1. Of note, (1) younger patients had a significantly lower risk (*P* = 0.002); (2) more tumours were located in the right colon in low-risk patients (*P* < 0.001); (3) the high-risk group contains more well differentiated tumours (*P* = 0.013); (4) the low-risk group had more cases of pT3–T4 TNM classifications (*P* = 0.044); (5) as the patient risk increased, the tumour sizes tended to decrease (*P *< 0.001); and (6) as fewer LNs were analysed the patient risk and overall mortality increased (*P* < 0.001 and *P *= 0.019, respectively). The following factors were identified as being related to OS (Table 2): age [hazard ratio (HR) = 1.05; 95% confidence intervals (CI) 1.03–1.07; *P* < 0.001], pT (HR = 1.79; 95% CI 1.44–2.22; *P* < 0.001), pN (HR = 1.22; 95% CI 1.10–1.35; *P* < 0.001), condensed TNM (HR = 1.55; 95% CI 1.27–1.90; *P* < 0.001), number of analysed LNs < 12 (HR = 0.74; 95% CI 0.56–0.98; *P* = 0.025), FEP (HR = 1.51; 95% CI 1.09–2.10; *P* = 0.019), chemotherapy (HR = 0.62; 95% CI 0.45–0.84; *P* = 0.014), locoregional recurrence (HR = 2.28; 95% CI 1.54–3.38; *P* < 0.001), and the presence of metastasis (HR = 3.51; 95% CI 2.66–4.65; *P* < 0.001). On the other hand, the following factors were identified as being related to DFS (Table 2): pT (HR = 1.87; 95% CI 1.38–2.53; *P* = 0.006), pN (HR = 1.89; 95% CI 1.49–2.41; *P* < 0.001), condensed TNM (HR = 1.89; 95% CI 1.41–2.52; *P* = 0.001), chemotherapy (HR = 1.93; 95% CI 1.33–2.81; *P* < 0.001), locoregional recurrence (HR = 12.28; 95% CI 8.13–15.54; *P* < 0.001), and the presence of metastasis (HR = 67.95; 95% CI 39.68–116.33; *P* < 0.001).

**Table 1:** Clinical and histopathological characteristics of 548 patients with colon cancer and the 346 patients with pN0 colon cancer within them grouped into 3 risk-groups according to their final error probability

Characteristic	Low-risk group (FEP < 2%)	Intermediate-risk group (FEP 2%–15%)	High-risk group (FEP > 15%)	Entire cohort	*P* value
Number of cases	35	244	67	548	
Age [years, median (range)]	66 (41–84)	74 (30–95)	76 (41–95)	72 (30–95)	0.002
Gender [cases (%)]					0.322
Male	12 (34.3%)	102 (41.8%)	33 (49.3%)	296 (54.0%)	
Female	23 (65.7%)	142 (58.2%)	34 (50.7%)	252 (46.0%)	
Tumour location [cases (%)]					0.001
Right colon	18 (51.4%)	108 (44.3%)	14 (20.9%)	224 (40.9%)	
Left colon	16 (45.7%)	118 (48.4%)	50 (74.6%)	290 (52.9%)	
Unknown	1 (2.9%)	18 (7.4%)	3 (4.5%)	34 (6.2%)	
Tumour size (mm, mean ± SD)	55 ± 30	48 ± 21	36 ± 19	46 ± 21	< 0.001
Histological classification [cases (%)]					0.344
Adenocarcinoma	29 (82.9%)	206 (84.4%)	62 (92.5%)	465 (84.9%)	
Mucinous variant	6 (17.1%)	33 (13.5%)	5 (7.5%)	74 (13.5%)	
Signet-ring cell	0	5 (2.0%)	0	9 (1.6%)	
Tumour differentiation grade [cases (%)]					0.013
Well	11 (31.4%)	70 (28.7%)	33 (49.3%)	151 (27.6%)	
Moderate	22 (62.9%)	160 (65.6%)	26 (38.8%)	343 (62.6%)	
Poor	1 (2.9%)	8 (3.3%)	5 (7.5%)	35 (6.4%)	
Unknown	1 (2.9%)	6 (2.5%)	3 (4.5%)	19 (3.5%)	
Lymphadenectomy					< 0.001
Number of analysed LNs [median (range)]	23 (19–45)	11 (6–18)	4 (1–5)	10 (1–45)	
< 12 analysed LNs [cases (%)]	0	142 (58.2%)	67 (100%)	308 (56.2%)	
≥ 12analysed LNs [cases (%)]	35 (100%)	102 (41.8%)	0	240 (43.8%)	
pT [6th edition, cases (%)]					0.044
pT1	1 (2.9%)	18 (7.4%)	12 (17.9%)	31 (5.7%)	
pT2	4 (11.4%)	47 (19.3%)	11 (16.4%)	85 (15.5%)	
pT3	28 (80%)	158 (64.8%)	34 (50.7%)	367 (67.0%)	
pT4	2 (5.7%)	20 (8.2%)	9 (13.4%)	63 (11.5%)	
pTx	0	1 (0.4%)	1 (1.5%)	2 (0.4%)	
pN [7th edition, cases (%)]					< 0.001
pN0	35 (100%)	244 (100%)	67 (100%)	346 (63.1%)	
pN+	0	0	0	202 (36.9%)	
pN1a	0	0	0	73 (13.3%)	
pN1b	0	0	0	70 (12.8%)	
pN2a	0	0	0	33 (6.0%)	
pN2b	0	0	0	26 (4.7%)	
Condensed TNM [7th edition, cases (%)]					0.094
I	5 (14.3%)	65 (26.6%)	23 (34.3%)	93 (17.0%)	
II	30 (85.7%)	179 (73.4%)	44 (65.7%)	253 (46.2%)	
III	0	0	0	202 (36.9%)	
Chemotherapy [cases (%)]					0.300
No	26 (74.3%)	197 (80.7%)	58 (86.6%)	371 (67.7%)	
Yes	9 (25.7%)	47 (19.3%)	9 (13.4%)	177 (32.3%)	
Overall mortality [cases (%)]					0.019
No	29 (82.9%)	162 (66.4%)	37 (55.2%)	334 (60.9%)	
Yes	6 (17.1%)	82 (33.6%)	30 (44.8%)	214 (39.1%)	
Overall recurrence [cases (%)]					0.185
No	33 (94.3%)	204 (83.6%)	54 (80.6%)	439 (80.1%)	
Yes	2 (5.7%)	40 (16.4%)	13 (19.4%)	109 (19.9%)	
Locoregional recurrence [cases (%)]					0.215
No	35 (100%)	224 (91.8%)	62 (92.5%)	509 (92.9%)	
Yes	0	20 (8.2%)	5 (7.5%)	39 (7.1%)	
Metastasis [cases (%)]					0.397
No	33 (94.3%)	214 (87.7%)	57 (85.1%)	456 (83.2%)	
Yes	2 (5.7%)	30 (12.3%)	10 (14.9%)	92 (16.8%)	
Follow-up [months, median (range)]	58 (3–95)	51 (1–95)	55 (1–91)	51 (1–99)	0.167

*FEP* final error probability, *SD* standard deviation, *LN* lymph node, *TNM* tumor-node-metastasis

**Table 2:** Univariate analysis of overall survival and disease-free survival according to clinical and histopathological characteristics

Characteristic	Overall survival		Disease-free survival	
	HR (95% CI)	*P* value	HR (95% CI)	*P* value
Age	1.05 (1.03–1.07)	< 0.001	1.00 (0.99–1.02)	0.456
Gender (female vs. male)	1.14 (0.87–1.49)	0.672	1.00 (0.69–1.46)	0.687
Location (left colon vs. right colon)	1.07 (0.80–1.40)	0.493	1.36 (0.91–2.04)	0.116
Histological classification (adenocarcinoma vs. mucinous variant vs. signet-ring cell)	1.12 (0.82–1.54)	0.227	1.25 (0.82–1.92)	0.180
Tumour differentiation grade (well vs. moderate vs. poor)	1.07 (0.87–1.33)	0.245	1.17 (0.87–1.59)	0.677
Tumour size	1.00 (0.99–1.01)	0.660	1.00 (0.99–1.01)	0.399
Lymphadenectomy				
Number of analysed LNs	0.97 (0.95–0.99)	0.073	0.99 (0.97–1.02)	0.990
< 12 analysed LNs (yes vs. no)	0.74 (0.56–0.98)	0.025	0.90 (0.61–1.31)	0.874
pT (6th edition, pT1 vs. pT2 vs. pT3 vs. pT4)	1.79 (1.44–2.22)	< 0.001	1.87 (1.38–2.53)	0.006
pN (7th edition, pN0 vs. pN1a vs. PN1b vs. pN2a vs. pN2b)	1.22 (1.10–1.35)	< 0.001	1.89 (1.49–2.41)	< 0.001
Condensed TNM (7th edition, stage I vs. stage II)	1.55 (1.27–1.90)	< 0.001	1.89 (1.41–2.52)	0.001
FEP	1.51 (1.09–2.10)	0.019	1.59 (0.98–2.59)	0.185
Chemotherapy (yes vs. no)	0.62 (0.45–0.84)	0.014	1.93 (1.33–2.81)	< 0.001
Locoregional recurrence (yes vs. no)	2.28 (1.54–3.38)	< 0.001	12.28 (8.13–15.54)	< 0.001
Metastasis (yes vs. no)	3.51 (2.66–4.65)	< 0.001	67.95 (39.68–116.33)	< 0.001

*HR* hazard ratio, *CI* confidence intervals, *ADC* adenocarcinoma, *LN* lymph node, *TNM* tumor-node-metastasis, *FEP* final error probability

**Fig. 2:**
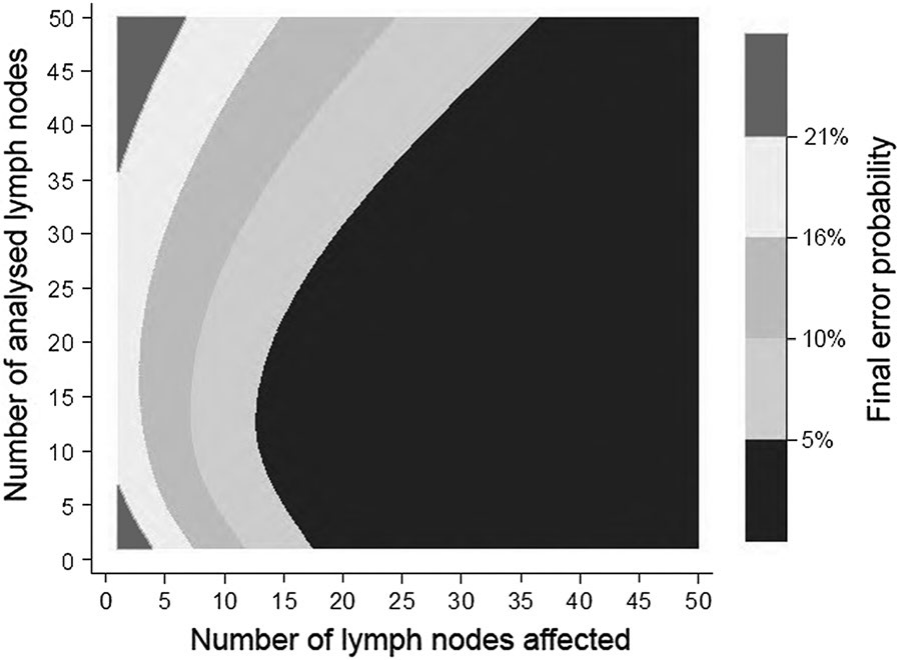
Contour plot of the final error probability in pN0 patient data shown in Table 3

Table 3 shows the FEP results according to the number of analysed LNs and is accompanied by a contour plot (Fig. 2) which represents its calculation graphically.

**Table 3:** Final error probability in pN0 patients adjusted to pN1 patients

Number of analysed lymph nodes	Final error probability (%)	Risk assessment
1	26.0	High risk
2	23.0	
3	20.0	
4	18.0	
5	16.0	
6	14.0	Intermediate risk
7	12.0	
8	10.0	
9	9.0	
10	8.0	
11	7.0	
12	6.0	
13	5.0	
14	4.0	
15	4.0	
16	3.0	
17	3.0	
18	2.0	
19	2.0	
20	2.0	
21	1.0	Low risk
22	1.0	
23	1.0	
24	1.0	
25	1.0	
26	1.0	
27	1.0	
28	0.5	
29	0.4	
30	0.3	
35	0.2	
40	0.1	
45	0	
50	0

**Fig. 3:**
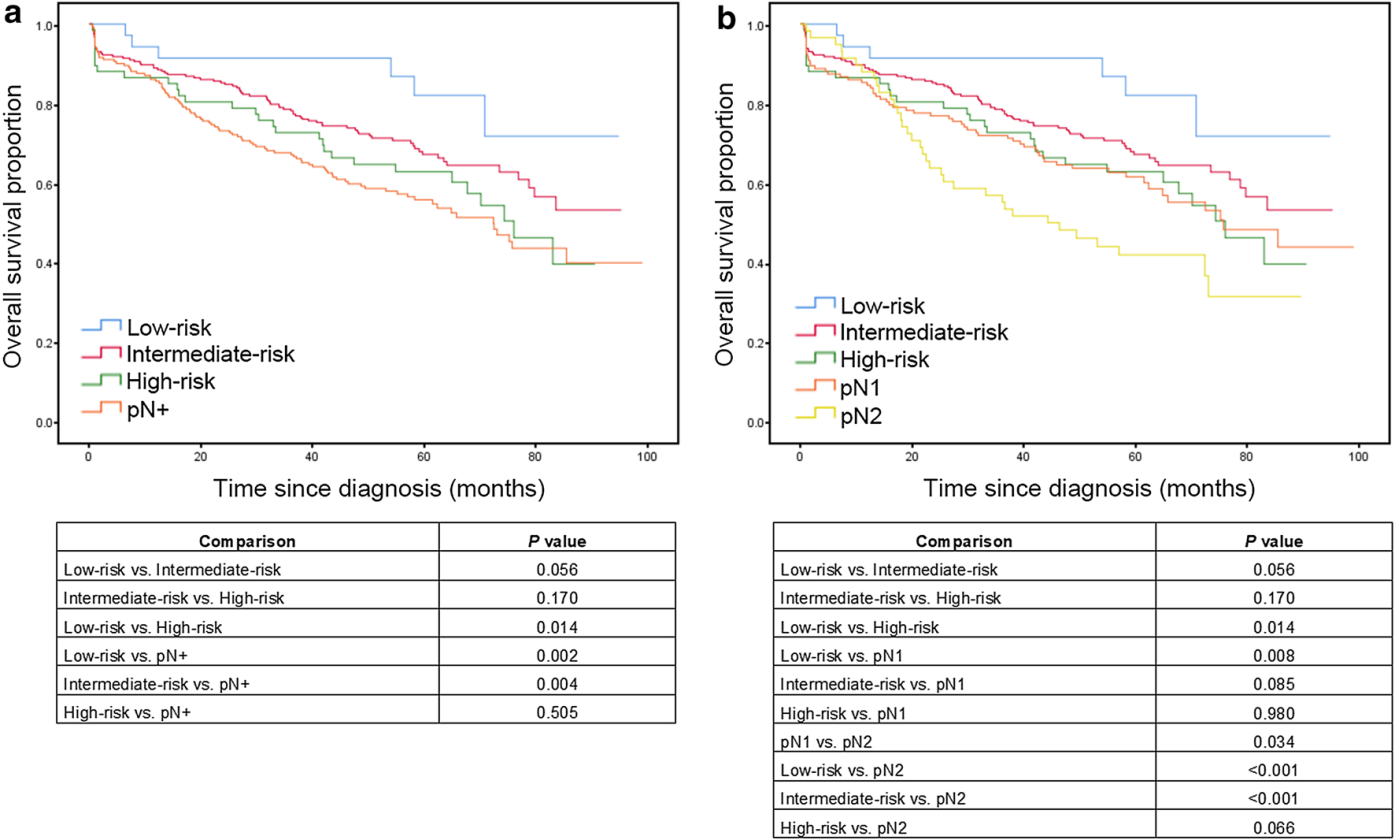
Overall survival according to final error probability (low-, intermediate- or high-risk groups) and pN stage (pN+ , pN1 or pN2). **a** Overall survival of low-, intermediate- and high-risk groups, and pN+ group. **b** Overall survival of low-, intermediate- and high-risk groups, pN1 and pN2 groups. P values obtained by log-rank test were summarized in the tables below

The OS charts (Fig. 3a) show that patients with LN involvement had the lowest OS rate than the three risk groups. This difference was statistically significant for the low- and intermediate-risk groups (*P* = 0.002 and *P* = 0.004, respectively), but not with respect to the high-risk group (*P *= 0.505). In other words, N0 patients with a high-risk FEP (> 15%) had an OS rate like that of pN+ patients.

**Fig. 4:**
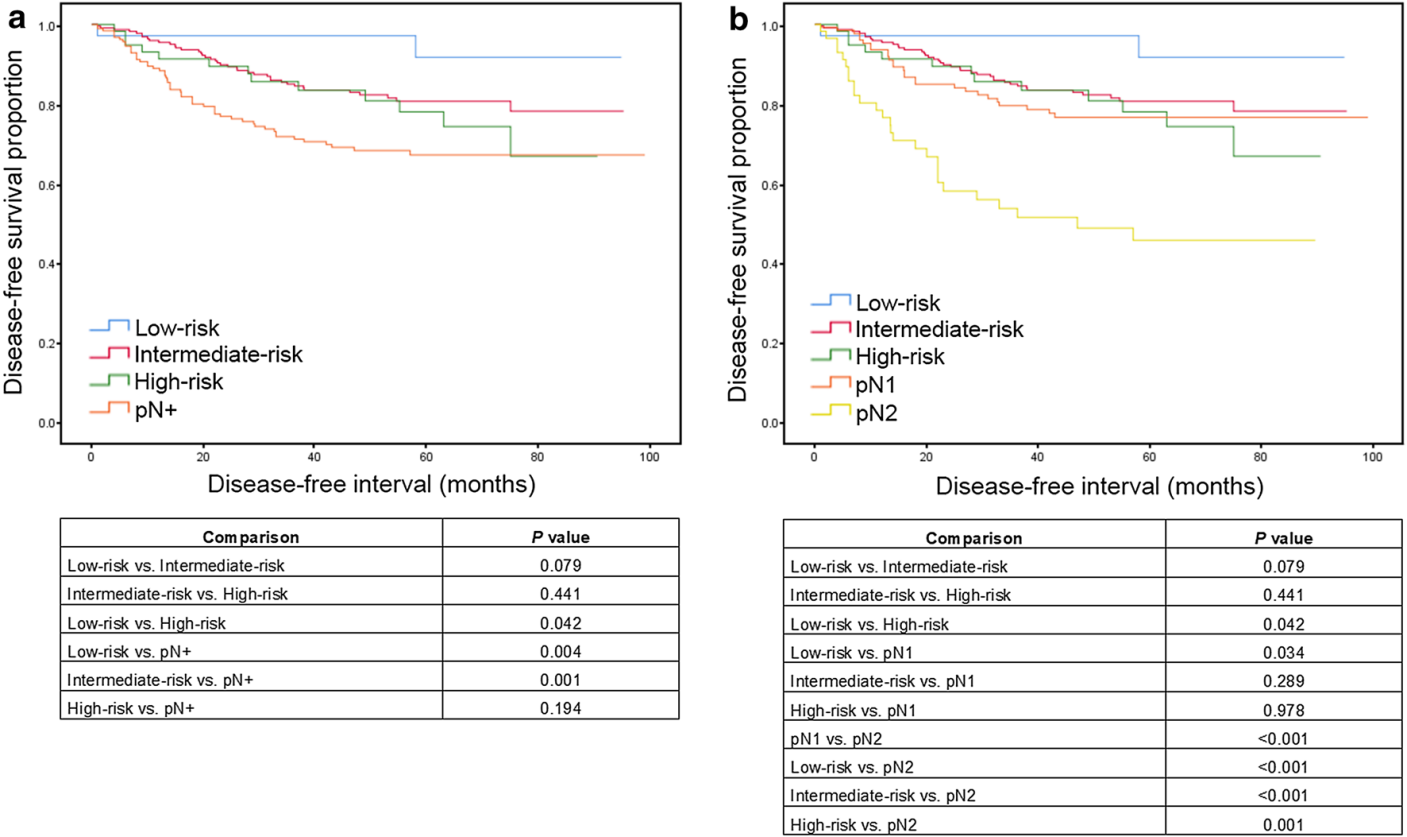
Disease-free survival according to final error probability (low-, intermediate- or high-risk groups) and pN stage (pN+ , pN1 or pN2). **a** Disease-free survival of low-, intermediate- and high-risk groups, and pN+ group. **b** Disease-free survival of low-, intermediate- and high-risk groups, pN1 and pN2 groups. P values obtained by log-rank test were summarized in the tables below

We subsequently carried out a similar analysis in which we divided patients with LN involvement into pN1 and pN2 groups. As shown in Fig. 3b, the OS rates differed between the low- and high-risk groups (*P* = 0.014). The pN1 group had statistically significant difference to the low-risk and pN2 group (*P *= 0.008 and P = 0.034, respectively). On the other hand, pN2 group had a significantly worse prognosis than low- and intermediate-risk groups (all *P* < 0.001), but there were no statistically significant differences between high-risk group (*P* = 0.066). Interestingly, the survival curves of the high-risk and pN1 patient were very similar (*P *= 0.980). In terms of DFS (Fig. 4a), the curves for the intermediate- and high-risk groups were similar until approximately 60 months’ follow-up (*P* = 0.906). After 80 months’ follow-up, the curve of high-risk group coincided with that of the pN+ group (P = 0.172). The curves for the intermediate- and high-risk group and pN1 groups were all similarly (Fig. 4b; intermediate-risk vs. high-risk, *P* = 0.441; intermediate-risk vs. pN1, *P* = 0.289; high-risk vs. pN1, *P* = 0.978).

## Discussion

LN involvement is one of the most important prognostic factors in colon cancer. Given its enormous importance, especially in terms of prognostic and therapeutic decisions, gaining a detailed picture of the true LN status of patients with colon cancer should be a priority for clinicians involved in the diagnostic–therapeutic process of these patients. In this sense, the use of FEP may be useful, especially for patient groups which more frequently see staging errors. Our group has extensive experience in using FEP to assess LN involvement in the contexts of colon [11], gastric [12], and breast cancers [13]. This method aims to calculate the probability that a pN+ patient in whom with insufficient analysed LNs will be erroneously classified as pN0. This has obvious clinical and prognostic consequences, especially in the light that stage III patients (i.e., those with LN involvement) can significantly benefit from chemotherapy [2, 14–16]. Correctly staging patients with colon cancer is very important given that around 60% of them do not currently present LN involvement at diagnosis [6, 7], a figure similar to the 63.3% staged as pN0 in this study.

FEP is particularly important for the patients in whom with insufficient analysed LNs because their TNM classification would not necessarily be accurate. This was the case in the series used in this study, in which the threshold 12 LNs were not analysed in 56% of the cases. Similar results have been reported in several previous studies, especially in population analyses like ours, in which this lymph-node threshold goal was not reached [2, 6, 17, 18]. There are likely several reasons why it is common for so few LNs to be analysed, likely because of factors related to the patient and surgeons, and to the type of anatomopathological study undertaken. Our results clearly show that FEP is strongly negatively related to lower OS rates (Fig. 3a). Moreover, patients with LN metastases have a poorer prognosis than those classified into the three risk groups. Most importantly, there were no statistically significant differences between the pN+ and high-risk patients. In other words, patients categorised into the high-risk FEP group had similar OS rates to those with LN involvement.

To try to increase the accuracy of our analysis, we further categorised pN1 and pN2 patients with LN infiltration and, as expected, pN2 patients had the poorest prognosis of the three groups whereas the low-risk group had the best OS rate (Fig. 3b). It is also important to highlight that the high-risk and pN1 patients had very similar OS curves. Therefore, the pN1 patients had a similar OS rate to the pN0 patients with a high staging error risk. In terms of DFS, patients with an intermediate and high risk had a DFS rate like that of pN1 patients (Fig. 4b). Thus, our data confirm that a minimum of 12 LNs must be analysed to reduce TNM classification staging errors. It should also be noted that low-risk patients had more right-colon neoplasms, were younger, and had larger tumours, i.e., their tumour characteristics favoured easier LN analysis [5, 19]. Similarly, when younger patients were included in this group and more of LNs were analysed, their OS rate was better.

The use of this mathematical model, which is based on the Bayes’ theorem, has been previously described in the literature in the identification of groups with similar prognoses from among patients with different cancers but with similar characteristics to those of our patient cohort. This model was first described by Kiricuta et al. [8] in 1992 and was applied in breast cancer to calculate the probability of tumour persistence after an incomplete axillary dissection, staging the patients according to the T category of the TNM classification. Later, Okamoto et al. [20] studied the probability of LN involvement in patients with negative sentinel-LN breast cancer using a Bayesian model. Iyer et al. [21] further demonstrated the usefulness of this method in 1652 patients with breast cancer in a study which aimed to evaluate the probability of LN involvement by staging it into T1 and T2. Following on from this work, Joseph et al. [22] studied 1585 patients with colorectal cancer and used this method to demonstrate the probability of LN involvement according to the number of analysed LNs using the same staging as Kiricuta et al. [8]. In a study of 480 patients with colon cancer, Martínez et al. [11] also showed that the risk of an erroneous negative ganglionic classification in colon cancer can be individualised by calculating its probability according to Bayes’ theorem.

According to the European Society for Medical Oncology guidelines, in our speciality a specific adjuvant treatment regimen is recommended for stage II patients with poor prognosis factors, including the analysis of fewer than 12 LNs. However, this regimen is not routinely applied, and the patient must sign their express informed consent to undergo this treatment [23].

It is useful to know the final probability of error in this field because it allows pN0 patients to be divided into several risk categories. This work shows that high-risk patients (patients without LN involvement but with five or fewer analysed LNs), have a similar prognosis to pN1 patients. The main limitations of this work lie in its retrospective non-randomised population study design which resulted in a loss of evidence. Another limitation was our use of the T category from the sixth rather than the seventh edition of the TNM classification; this was because this version was in effect during the period in which the study data was collected. However, to help reduce this bias, we allocated the patients either into a group with earlier-stage tumours (T1 and T2) or into one with locally more advanced tumours (stages T3 and T4). This minimised the effect of any modifications to the T factor classification made between these two TNM editions.

## Conclusions

The application of Bayes’ theorem in the calculation of FEP is useful to delimit risk subgroups from among patients without LN involvement. Therefore, the FEP system complements the TNM LN staging classification well by improving the discrimination of the colon cancer prognosis in pN0 patients with a high-risk of having been misclassified as free of LN involvement because too few of their LNs had been analysed.

## Data Availability

The primary dataset will not be shared because it is subject to confidentiality restrictions.

## Abbreviations

FEP: final error probability
OS: overall survival
TNM: tumor-node-metastasis
DFS: disease-free survival
HR: hazard ratio
CI: confidence intervals
SD: standard deviation
CUSUM: cumulative sum
